# Supplementary material for: Enabling precision medicine via standard communication of HTS provenance, analysis, and results
Source: PLoS Biol. 2018 Dec 31;16(12):e3000099. doi: 10.1371/journal.pbio.3000099 (PMC6338479; doi:10.1371/journal.pbio.3000099)
Supplement: S1 Text — (DOCX) [file pbio.3000099.s001.docx]

**Supplementary File 1.** The example BCO, and some additional details on projects, and concepts mentioned in the main text of the publication.

Example BioCompute for HCV1a ledipasvir resistance SNP detection.

{

"bco_id": "https://w3id.org/biocompute/1.3.0/examples/HCV1a.json",

"checksum": "06DACE70679F35BA87A3DD6FFFED4ED24A4F5B8C2571264C37E5F1B3ADE04A31",

"bco_spec_version" : "https://w3id.org/biocompute/1.3.0/",

"provenance_domain": {

"name": "HCV1a ledipasvir resistance SNP detection",

"version": "2.9",

"review": [

{

"status": "approved",

"reviewer_comment": "Approved by GW staff",

"date": "2017-11-12T12:30:48-0400",

"reviewer": {

"name": "Charles Hadley King",

"affiliation": "George Washington University",

"email": "hadley_king@gwu.edu",

"contribution": ["curatedBy"],

"orcid": "https://orcid.org/0000-0003-1409-4549"

}

},

{

"status": "approved",

"reviewer_comment": "The revised BCO looks fine",

"date": "2017-12-12T12:30:48-0400",

"reviewer": {

"name": "Eric Donaldson",

"affiliation": "FDA",

"email": "Eric.Donaldson@fda.hhs.gov",

"contribution": ["curatedBy"]

}

}

],

"obsolete_after": "2118-09-26T14:43:43-0400",

"embargo": {

"start_time": "2000-09-26T14:43:43-0400",

"end_time": "2000-09-26T14:43:45-0400"

},

"created": "2017-01-24T09:40:17-0500",

"modified": "2018-09-21T14:06:14-0400",

"contributors": [

{

"name": "Charles Hadley King",

"affiliation": "George Washington University",

"email": "hadley_king@gwu.edu",

"contribution": ["createdBy", "curatedBy"],

"orcid": "https://orcid.org/0000-0003-1409-4549"

},

{

"name": "Eric Donaldson",

"affiliation": "FDA",

"email": "Eric.Donaldson@fda.hhs.gov",

"contribution": ["authoredBy"]

}

],

"license": "https://spdx.org/licenses/CC-BY-4.0.html"

},

"usability_domain": [

"Identify baseline single nucleotide polymorphisms (SNPs)[SO:0000694], (insertions)[SO:0000667], and (deletions)[SO:0000045] that correlate with reduced (ledipasvir)[pubchem.compound:67505836] antiviral drug efficacy in (Hepatitis C virus subtype 1)[taxonomy:31646]",

"Identify treatment emergent amino acid (substitutions)[SO:1000002] that correlate with antiviral drug treatment failure",

"Determine whether the treatment emergent amino acid (substitutions)[SO:1000002] identified correlate with treatment failure involving other drugs against the same virus",

"GitHub CWL example: https://github.com/mr-c/hive-cwl-examples/blob/master/workflow/hive-viral-mutation-detection.cwl#L20"

],

"extension_domain":{

"fhir_extension": [

{

"fhir_endpoint": "http://fhirtest.uhn.ca/baseDstu3",

"fhir_version": "3",

"fhir_resources": [

{

"fhir_resource": "Sequence",

"fhir_id": "21376"

},

{

"fhir_resource": "DiagnosticReport",

"fhir_id": "6288583"

},

{

"fhir_resource": "ProcedureRequest",

"fhir_id": "25544"

},

{

"fhir_resource": "Observation",

"fhir_id": "92440"

},

{

"fhir_resource": "FamilyMemberHistory",

"fhir_id": "4588936"

}

]

}

],

"scm_extension": {

"scm_repository": "https://github.com/example/repo1",

"scm_type": "git",

"scm_commit": "c9ffea0b60fa3bcf8e138af7c99ca141a6b8fb21",

"scm_path": "workflow/hive-viral-mutation-detection.cwl",

"scm_preview": "https://github.com/example/repo1/blob/c9ffea0b60fa3bcf8e138af7c99ca141a6b8fb21/workflow/hive-viral-mutation-detection.cwl"

}

},

"description_domain": {

"keywords": [

"HCV1a",

"Ledipasvir",

"antiviral resistance",

"SNP",

"amino acid substitutions"

],

"xref": [

{

"namespace": "pubchem.compound",

"name": "PubChem-compound",

"ids": ["67505836"],

"access_time": "2018-13-02T10:15-05:00"

},

{

"namespace": "pubmed",

"name": "PubMed",

"ids": ["26508693"],

"access_time": "2018-13-02T10:15-05:00"

},

{

"namespace": "so",

"name": "Sequence Ontology",

"ids": ["SO:000002", "SO:0000694", "SO:0000667", "SO:0000045"],

"access_time": "2018-13-02T10:15-05:00"

},

{

"namespace": "taxonomy",

"name": "Taxonomy",

"ids": ["31646"],

"access_time": "2018-13-02T10:15-05:00"

}

],

"platform": ["HIVE"],

"pipeline_steps": [

{

"step_number": 1,

"name": "HIVE-hexagon",

"description": "Alignment of reads to a set of references",

"version": "1.3",

"prerequisite": [

{

"name": "Hepatitis C virus genotype 1",

"uri": {

"uri": "http://www.ncbi.nlm.nih.gov/nuccore/22129792",

"access_time": "2017-01-24T09:40:17-0500"

}

},

{

"name": "Hepatitis C virus type 1b complete genome",

"uri": {

"uri": "http://www.ncbi.nlm.nih.gov/nuccore/5420376",

"access_time": "2017-01-24T09:40:17-0500"

}

},

{

"name": "Hepatitis C virus (isolate JFH-1) genomic RNA",

"uri": {

"uri": "http://www.ncbi.nlm.nih.gov/nuccore/13122261",

"access_time": "2017-01-24T09:40:17-0500"

}

},

{

"name": "Hepatitis C virus clone J8CF, complete genome",

"uri": {

"uri": "http://www.ncbi.nlm.nih.gov/nuccore/386646758",

"access_time": "2017-01-24T09:40:17-0500"

}

},

{

"name": "Hepatitis C virus S52 polyprotein gene",

"uri": {

"uri": "http://www.ncbi.nlm.nih.gov/nuccore/295311559",

"access_time": "2017-01-24T09:40:17-0500"

}

}

],

"input_list": [

{

"uri": "http://example.com/dna.cgi?cmd=objFile&ids=514683",

"access_time": "2017-01-24T09:40:17-0500"

},

{

"uri": "http://example.com/dna.cgi?cmd=objFile&ids=514682",

"access_time": "2017-01-24T09:40:17-0500"

}

],

"output_list": [

{

"uri": "http://example.com/data/514769/allCount-aligned.csv",

"access_time": "2017-01-24T09:40:17-0500"

}

]

},

{

"step_number": 2,

"name": "HIVE-heptagon",

"description": "variant calling",

"version": "1.3",

"input_list": [

{

"uri": "http://example.com/data/514769/dnaAccessionBased.csv",

"access_time": "2017-01-24T09:40:17-0500"

}

],

"output_list": [

{

"uri": "http://example.com/data/514801/SNPProfile.csv",

"access_time": "2017-01-24T09:40:17-0500"

},

{

"uri": "http://example.com/data/14769/allCount-aligned.csv",

"access_time": "2017-01-24T09:40:17-0500"

}

]

}

]

},

"execution_domain": {

"script":[

{

"uri": {

"uri": "https://example.com/workflows/antiviral_resistance_detection_hive.py"

}

}

],

"script_driver": "shell",

"software_prerequisites": [

{

"name": "HIVE-hexagon",

"version": "babajanian.1",

"uri": {

"uri": "http://example.com/dna.cgi?cmd=dna-hexagon&cmdMode=-",

"access_time": "2017-01-24T09:40:17-0500",

"sha1_chksum": "d60f506cddac09e9e816531e7905ca1ca6641e3c"

}

},

{

"name": "HIVE-heptagon",

"version": "albinoni.2",

"uri": {

"uri": "http://example.com/dna.cgi?cmd=dna-heptagon&cmdMode=-",

"access_time": "2017-01-24T09:40:17-0500"

}

}

],

"external_data_endpoints": [

{

"name": "HIVE",

"url": "http://example.com/dna.cgi?cmd=login"

},

{

"name": "access to e-utils",

"url": "http://eutils.ncbi.nlm.nih.gov/entrez/eutils/"

}

],

"environment_variables": {

"HOSTTYPE": "x86_64-linux",

"EDITOR": "vim"

}

},

"parametric_domain": [

{"param": "seed", "value": "14", "step": "1"},

{"param":"minimum_match_len", "value": "66", "step": "1"},

{"param": "divergence_threshold_percent", "value": "0.30", "step": "1"},

{"param": "minimum_coverage", "value": "15", "step": "2"},

{"param": "freq_cutoff", "value": "0.10", "step": "2"}

],

"io_domain": {

"input_subdomain": [

{

"uri": {

"filename": "Hepatitis C virus genotype 1",

"uri": "http://www.ncbi.nlm.nih.gov/nuccore/22129792",

"access_time": "2017-01-24T09:40:17-0500"

}

},

{

"uri": {

"filename": "Hepatitis C virus type 1b complete genome",

"uri": "http://www.ncbi.nlm.nih.gov/nuccore/5420376",

"access_time": "2017-01-24T09:40:17-0500"

}

},

{

"uri": {

"filename": "Hepatitis C virus (isolate JFH-1) genomic RNA",

"uri": "http://www.ncbi.nlm.nih.gov/nuccore/13122261",

"access_time": "2017-01-24T09:40:17-0500"

}

},

{

"uri": {

"uri": "http://www.ncbi.nlm.nih.gov/nuccore/386646758",

"access_time": "2017-01-24T09:40:17-0500"

}

},

{

"uri": {

"filename": "Hepatitis C virus S52 polyprotein gene",

"uri": "http://www.ncbi.nlm.nih.gov/nuccore/295311559",

"access_time": "2017-01-24T09:40:17-0500"

}

},

{

"uri": {

"filename": "HCV1a_drug_resistant_sample0001-01",

"uri": "http://example.com/nuc-read/514682",

"access_time": "2017-01-24T09:40:17-0500"

}

},

{

"uri": {

"filename": "HCV1a_drug_resistant_sample0001-02",

"uri": "http://example.com/nuc-read/514683",

"access_time": "2017-01-24T09:40:17-0500"

}

}

],

"output_subdomain": [

{

"mediatype": "text/csv",

"uri": {

"uri": "http://example.com/data/514769/dnaAccessionBased.csv",

"access_time": "2017-01-24T09:40:17-0500"

}

},

{

"mediatype": "text/csv",

"uri": {

"uri": "http://example.com/data/514801/SNPProfile*.csv",

"access_time": "2017-01-24T09:40:17-0500"

}

}

]

},

"error_domain": {

"empirical_error": {

"false_negative_alignment_hits": "<0.0010",

"false_discovery": "<0.05"

},

"algorithmic_error": {

"false_positive_mutation_calls_discovery": "<0.00005",

"false_discovery": "0.005"

}

}

}

## **BioCompute Objects (BCOs)**

BioCompute Objects (BCOs) were conceptualized to harmonize NGS computational results and data formats and encourage interoperability which is useful for FDA submissions[[1](#_ENREF_1)]. Each BCO comprises information on the arguments and versions of executable programs in a pipeline, references to input/output data, a usability domain, keywords, a list of authors, and other important sources of metadata. The conceptual schema for BCO creation is built on top of two layers: the data definition framework and the BCO framework (see technical specifications document at https://github.com/biocompute-objects/BCO_Specification).

# The data definition framework contains primitive data type definitions. Such data types cannot be deconstructed any further without losing meaning or some other important information, like an integer or character. Complex types are composed of multiple primitive types or even multiple complex types, like a character string. Using these principles, one can construct a datum that has the ability to represent any level of complexity needed, with the only constraint being the amount of available storage memory or computing power.

When defining a field in a data type, one can place any number of constraints on the data that the field will accept as valid. If one were constructing a data type field to hold DNA sequencing information, one could restrain the type of characters that field would accept. This further refinement ensures that only the characters used to represent nucleic acids would be accepted as input in this field (A, T, C, and G).

The second framework layer defines a derived data type. Extending the same principles that allowed one to construct a string representing a DNA sequence from the primitive character type, it is possible to construct a data type definition with the absolute minimum fields necessary to create a BCO. By taking the primitive BioCompute object type and adding parametric and metadata fields unique to a particular instance, one can get the final, unique BCO for the specified workflow and analysis.

The declarative nature of BCOs suggests an implementation with minimal procedural barriers. Though not a requirement, the use of schema-less representation in JSON format that does not impede the identification of a validating schema accords with the purpose of Compute Objects such as BCOs and FHIR I/O. For an example of editable BCO objects, see<https://mathbiol.github.io/bco>. Accessing experimental data and their origin is challenging; thus, aligning frameworks that encourage interoperability such as Data Tag Suite (DATS) help attain data standards that are easily verifiable, discoverable, reusable, and interoperable[[2](#_ENREF_2)]. DATS is a mechanism that enables the data to be easily searchable, findable, and reusable. The BCO takes a snapshot of the whole experiment computational procedure where the input data are provided and described in detail along with all the default and experimental procedures used in the dataset. The output domain of the BCO includes the results from the experiment in the dataset so that any other user can run the exact experiment and produce the same results. The BCO captures curated ontologies which are in reviewed and highly maintained databases to ensure that they are easily accessible and searchable.

The BCO can serve as an umbrella of standards allowing for standards such as Common Workflow Language (CWL), Fast Healthcare Interoperability Resources (FHIR), Global Alliance for Genomics and Health (GA4GH), and Research Objects (RO) to be embedded within BCO fields. Enabling BCOs to incorporate existing standards provides a universal framework for including existing advances in workflow and data specifications that increase the specificity for describing a workflow and the related provenance. Moreover, the umbrella approach also supports a minimal effort form-based BCO that can be quickly implemented, allowing for a rapid initial implementation which can evolve over time to capture the greater specificity made available by incorporating existing standards.

## **Workflow Management Systems**

*bcbio-nextgen[*[*3*](#_ENREF_3)*]* [<https://github.com/chapmanb/bcbio-nextgen>] has a domain-specific language for executing pipelines in NGS analysis, in particular, variant calling like RNA-seq and small RNA analysis. Unlike other systems, bcbio-nextgen focuses on the parameters of the pipeline and a choice of algorithms, rather than the declaration of the steps and their underlying command lines. Bcbio handles installation of all third-party tools and reference datasets required for its pipelines. Pipelines can be executed using multiple cores or parallel messaging on a cluster environment, which can facilitate high-performance schedulers like LSF and SGE.

*Snakemake[*[*4*](#_ENREF_4)*]* [[doi:10.1093/bioinformatics/bts480](https://doi.org/10.1093/bioinformatics/bts480)] is a declarative Python-like workflow language similar to a traditional *Makefile*. Snakemake files contain rules on how to create a particular file by executing a command or script and declaring which other files or file patterns the rule depend on, thus implicitly containing the rule execution order. The integration with Python simplifies “shim” operations between steps (e.g., handling different genomics file formats). The resulting workflow can be effectively executed on a local single-core machine, a multi-core server, or scaled to compute-clusters of different architectures.

*Nextflow[*[*5*](#_ENREF_5)*]* [[doi:10.1038/nbt.3820](https://doi.org/10.1038/nbt.3820)] is a Python-like language for data-driven computational bioinformatics pipelines, with a strong focus on reproducibility and scalability. Nextflow uses Docker [<https://www.docker.com/>] to containerize and deploy the third-party tools the workflow relies on. A Nextflow workflow is declared by defining processes, which consume and produce messages on asynchronous channels. Channels are then wired together to form a workflow, which can be executed efficiently on a multitude of HPC and cloud platforms, including SGE, LSF, SLURM, Apache Ignite, and Kubernetes.

*Toil[*[*6*](#_ENREF_6)*]* [[doi:10.1038/nbt.3772](https://doi.org/10.1038/nbt.3772)] can run large-scale scientific workflows on cloud and HPC environments defined in either Common Workflow Language (CWL), Workflow Description Language (WDL), or Python Toil scripts. Toil jobs can be containerized using Docker and executed on multiple cloud environments (like AWS, Microsoft Azure, Google Cloud), in HPC environments using Grid Engine, or on distributed systems using Apache Mesos, with a strong emphasis on scalability and portability.

## **Bioinformatics Platforms**

### *DNAnexus:* Founded in 2009, DNAnexus[[7](#_ENREF_7)] (www.dnanexus.com) is a global, cloud-based platform for genomic data analysis and management. To meet increasing demands for efficient DNA data organization, DNAnexus arose as a tool for quick analysis of innumerable raw sequencing data, secure integration of genomic data with clinical infrastructures, and increased collaboration among scientists. The platform allows users to custom, port, and reproduce pipelines to the cloud-based infrastructure, making the data easily accessible. DNAnexus ensures clinically compliant data is secure and auditable. Additionally, DNAnexus facilitates collaboration among colleagues and upstream/downstream partners, easing data sharing.

### *Galaxy:* Started in 2005, Galaxy[[8](#_ENREF_8)] (<https://galaxyproject.org/>) is an open-source, web-based platform that enables scientists without informatics expertise to perform computational analysis through the web[[9](#_ENREF_9)]. Existing analysis tools are integrated into Galaxy and are available through the consistent web interface that can be deployed on any Unix system. Because the Galaxy software is highly customizable, the platform integrates with a wide variety of compute environments, making data processing accessible among users. Automated, multi-step analyses can be performed by combining tools into workflows (pipelines), and all analyses are reproducible[[10](#_ENREF_10)]. By bridging the gap between tool developers and scientists, Galaxy helps both constituencies accelerate their research. The Public Galaxy Server (<https://usegalaxy.org/)> is an installation of the Galaxy software combined with many common analysis tools, workflows, and data sources. A free resource, the site provides substantial compute resources to analyze large datasets, transforming data to reproducible formats. The Galaxy Tool Shed (<https://usegalaxy.org/toolshed)> facilitates sharing of Galaxy tools as a central location where developers can upload their tool configurations, allowing greater collaboration for computational analyses. Galaxy formats data to be stored, imported, and exported for analyses and open workflows. Galaxy predates the implementation of community standards like GA4GH schemas, CWL, and BCO, so the platform provides limited support for data standardization. Future developments should standardize Galaxy’s data and methods to comply with current community standards.

### *HIVE (High-performance Integrated Virtual Environment):* The HIVE[[11](#_ENREF_11),[12](#_ENREF_12)] platform is a cloud infrastructure that hosts a web-accessible interface that allows users to interact (deposit, share, retrieve, annotate, compute, visualize) with large volumes of NGS data. User interaction is conducted in a scalable fashion through the platform’s connected distributed storage library and distributed computational resources. A novel aspect of HIVE compared to existing technologies is the seamless integration of tools and data, hierarchical sharing, secure object traceability and auditing. HIVE allows users to regulate, reproduce, share and access data, and store computational workflows, complete with input/output data, parameters, versions, and tool.

#

### *Seven Bridges Genomics (SBG):* Seven Bridges is a cloud-based platform that enables rapid and collaborative analysis of datasets in concert with other forms of biomedical data by utilizing High Throughput Sequencing (HTS) technologies. To interpret specifications, workflow engines, like Reproducible Analyses for Bioinformatics (Rabix) Executor. enable reproducibility by making data processing easier. Rabix, an open-source CWL executor, is embedded within the platform and orchestrates multi-instance and parallelizable execution on AWS and Google (http://rabix.org). The Rabix Composer, an integrated development environment for CWL, allows workflows to be constructed and executed locally and readily deployed on the platform, furthering interoperability. Seven Bridges Core Infrastructure enables standardized data analysis and collaboration support, as exemplified by Cavatica. Cavatica allows physicians to share and analyze genomic profiles of pediatric brain tumors when deciding on clinical treatment plans. Cavatica exemplifies the applications of reproducible data, allowing greater collaboration and treatment efficiency.

### **National Cancer Institute (NCI) Cloud Resources:**

The NCI Cloud Resources were formerly known as NCI Cancer Genomics Cloud (CGC) Pilots, which were conceptualized in 2013 to democratize access to NCI-generated genomic data and facilitate analysis. Three Cloud Pilot awardees—the Broad Institute (<https://software.broadinstitute.org/firecloud/>), the Institute for Systems Biology (<http://cgc.systemsbiology.net/>), and Seven Bridges (<http://www.cancergenomicscloud.org/>) have independently developed cloud-based analysis platforms. As a Software-as-a-Service built on commercial cloud architectures, these cloud resources offer researchers the flexibility to utilize their own tools in the form of Docker containers. Tools can also be joined to form complex workflows described by Common Workflow Language (CWL) or Workflow Description Language (WDL). In a user-friendly graphical user interface, computation and data are encapsulated in a secured, access-controlled environment that also allows for sharing with collaborators.

**Internet2 Community**

Internet2, the U.S. research and education network, connects academic, government (including NIH, FDA, and CDC), and life sciences companies. Internet2 also extends connectivity to the local level, including many healthcare institutions, through its high bandwidth U.S. Unified Community Anchor Network (U.S. UCAN) Program. Together these members constitute a diverse problem-solving community that can share data frictionlessly at high speeds. Finally, over six million users at member institutions collaborate using Internet2’s InCommon trust and federated identity management system. This enables virtualization of compute and storage resources, both private and cloud, to reduce costs and speed of both information sharing and discovery. As this virtual infrastructure becomes more intelligently responsive to data-driven operations, the BCO initiative promises to improve data findability and execution of distributed workflows through enhanced structuring of data.

## **The Common Workflow Language (CWL)**

Common Workflow Language (CWL)[[13](#_ENREF_13)] is an open community-led standard to describe workflow and tools for data-intensive sciences (including Bioinformatic and Medical Imaging analyses) with a strong focus on reproducibility, reusability, scalability, and portability. CWL files can be executed by multiple workflow engine implementations, including Toil, Arvados, and Rabix Bunny[[14](#_ENREF_14)]. These implementations again support execution locally, on clusters, and on multiple cloud and HPC environments.

In an effort to standardize, CWL has focused on the current ability of most workflow systems: *Execute command line tools* and *coordination of their inputs and outputs* in a top-to-bottom pipeline. At the heart of CWL workflows are WL tool descriptions. A command line, often with an accompanying Docker container, is described with parameters and linkage to and from registries like ELIXIR’s (European Life-sciences Infrastructure for Biological Information) bio.tools [[15](#_ENREF_15)]. These are then wired together in another YAML file to form a workflow template, which can be executed repeatedly on any supported platform by specifying input files and workflow parameters.

CWL allows scientists to express their data and workflows in a universal computational language, generating greater method reproducibility for the genomic community. A community-specific computing language builds standardization from the data producer, avoiding the “Tower of Babel” issue of varied languages causing miscommunication. CWL lays the foundation for expression of BCOs, inherently embedding reproducibility in the BCO specification.

## **Fast Healthcare Interoperability Resources (FHIR)**

FHIR is an all-encompassing standard for communicating clinical and health information. As such, it includes genomic components known as FHIR Genomics API/specification integrated in its core. These genomics components evolved from the SMART on FHIR Genomics standard[[16](#_ENREF_16)] and integrated work of the HL7 Clinical Genomics Workgroup, and the standard is based on the requirements of Meaningful Use 3.0. FHIR is an emerging standard for electronic medical records (EMRs) and clinical apps being adopted by numerous vendors in the healthcare space. Projects based on FHIR Genomics enable lab vendors to share clinical genomic information for precision medicine and EMR-based patient information for research studies, such as the NIH’s All of Us program. Projects based on FHIR enable both the data and ecosystem to exist for communication of clinical and genomic information on individual patients.

Capturing genomic provenance information via FHIR enables clinical trials, research, and clinical interpretations to be traceable back to the original methods, workflows, and parameters used. This, in turn, facilitates robust and reproducible clinical interpretations of genomics and comparisons to be made across patients in which similar methods were used. FHIR utilizes the PROV standard introduced earlier to capture provenance information. Practically applied, a clinical genomic sequence entity target can be generated via a particular workflow instance activity through a specific laboratory agent. As part of the FHIR Release 3 API/specification, provenance examples are constructed that enable the capture of workflows via CWL and workflow instance for potential FDA submissions via BCO. FHIR equips clinicians, researchers, and regulators to be able to trace, reproduce, and reinterpret/compare genomic data[[17](#_ENREF_17)]. By communicating clinical information, FHIR lays the groundwork for collaboration in BCO implementation, permitting easy data sharing.

## **Global Alliance for Genomic Health (GA4GH)**

The Global Alliance for Genomic Health (GA4GH) is a cooperative framework established as a resource for genomic research and phenotype sharing [[18](#_ENREF_18)]. GA4GH was created as a common framework to enable responsible, voluntary, and secure sharing of data to advance precision care[[19](#_ENREF_19)]. It has faced challenges in data aggregation procedures, but has demonstrated the potential of a synergistic data sharing culture. To execute data sharing goals, GA4GH schemas, which define how to access genomic data, and APIs, which implement these schemas, have been created. These schemas facilitate DNA sequence data exchange and use common, user-friendly web protocols to overcome incompatible infrastructures [[18](#_ENREF_18)]. An application of GA4GH is the BRCA Exchange (<http://brcaexchange.org/>), which provides a searchable resource that combines breast cancer-contributing germline variants from eight different institutions. Overall, GA4GH is not intended to enforce data standards, but rather provides recommendations to influence and persuade the advantages of a collaborative data culture[[20](#_ENREF_20)]. GA4GH enables researchers to communicate their data to clinicians and the FDA. Together with FHIR, GA4GH allows BCOs to be utilized in clinical and basic research, and as BCOs are integrated with these specifications, data communication and provenance information become interlinked.

## **Research Objects (ROs)**

Research Objects [[http://ResearchObject.org/](http://researchobject.org/)] is a new publication model that improves reproducibility of scientific data by capturing provenance, quality, credit, attribution, and methods [[21](#_ENREF_21),[22](#_ENREF_22)]. A Research Object (RO) is an aggregation mechanism that bundles the method of a computational analysis (e.g., expressed as scripts and workflows) and all associated materials, metadata, and annotations using existing Linked Data standards[[23](#_ENREF_23)]. ROs consist of a container of files with a manifest to provide meaningful information about what those files are, what they mean, how they relate and provide provenance and versioning information [11]. The containers vary, such as *Docker*, *BagIt[*[*24*](#_ENREF_24)*]* or the Zip Archive *Research Object Bundle* [<https://w3id.org/bundle/>]. Resource content can be embedded or referenced externally using URIs, which may require further authentication and allows for greater regulation. ROs collect the general data and workflow provenance necessary for reproducibility, acting as a lab notebook for computational processes.

ROs have been applied to improve reproducibility of workflows [[25](#_ENREF_25)] and to describe large datasets [[24](#_ENREF_24),[26](#_ENREF_26)]. By its aggregating nature, ROs go beyond the experimental description to bring together the wider digital context and conduct of scientific processes, including input/output data, methods, software, actors, analysis, dissemination, sharing, reuse, and the links/relationships between these gathered resources[[27](#_ENREF_27)].

The *wf4ever* project[[28](#_ENREF_28)], which primarily developed the RO model[[29](#_ENREF_29)], specified a workflow description vocabulary (*wfdesc*) [<https://w3id.org/ro/2016-01-28/wfdesc>] that defines resources associated with a workflow specification within a Research Object Framework. The workflow description vocabulary defines three main terms: workflow as a process node and data link, process as a software tool that executes specific actions, and data link as a tool used to encode dependencies between computational nodes. This vocabulary is the basis for the CWL specification[[13](#_ENREF_13)]: CWL describes the workflow and its means of execution, whereas the RO provides the description relating the workflow to its provenance, purpose, and so forth. The PROV resource (Figure 2) ontology is also the basis of the RO workflow provenance model *wfprov* [<https://w3id.org/ro/2016-01-28/wfprov>], linking the various specifications (CWL, FHIR, ROs) under a similar basis that provides interoperability leveraged by BCOs.

## **Journal/Peer Review Perspective**

The genomic community has come to acknowledge the necessity of data sharing and communication to facilitate reproducibility, standardization, and provenance, reshaping the way research is conducted, ensuring openness and maximum benefit by the scientific community who ultimately is the consumer of the products of a research publication[[30](#_ENREF_30)].

This issue is clearly exemplified by the lack of interoperability between the web service interfaces of major bioinformatics centers, including the National Center for Biotechnology Information (NCBI), the European Bioinformatics Institute (EBI) in the UK, DNA Data Bank of Japan (DDBJ)/Kyoto Encyclopedia of Genes, and Genomes (KEGG)/Protein Data Bank Japan (PDBj) in Japan. As the centers’ web service models are all based on open standards, their databases and computational resources are expected to be interoperable[[31](#_ENREF_31)]. Despite the large amount of data in these services, these centers use their own data type definitions, making it harder for end users and developers to utilize these services to create biological analysis workflows[[32](#_ENREF_32)].

While lack of interoperability is not uncommon in computational biology, significant efforts have been made to increase interoperability between web services, standardize exchangeable data types, and adopt compatible interfaces for each service[[33](#_ENREF_33)]. Several projects and workshops have already begun progress to bridge the gap: the BioMoby project defined ontologies for data types and methods used in its services, and it provides a centralized repository for its service discovery[[34](#_ENREF_34)]; Open Bio* libraries have been developed for the major computing languages (i.e., Perl, Python, Ruby, and Java) to maximize bioinformatics web services and to create collaborative compatible data models for common biological objects[[35](#_ENREF_35)]; the EDAM ontology of bioinformatics operations, types of data and identifiers, topics and formats used by CWL and workflow ROs[[36](#_ENREF_36)], the DBCLS BioHackathon improves web service interoperability and collaboration between major database centers[[33](#_ENREF_33)]; and the HTS-CSRS (BioCompute) Workshop hosted by GW and the FDA is a cross-disciplinary endeavor emphasizing standardization of data storage and collection, communication of these genetic data, and the necessity of reproducibility of these analyses to ensure their potential clinical applications[[37](#_ENREF_37)]. These are just a few examples of the efforts that combine technologies, ontologies, and standards to enhance computational analysis information. The FAIRsharing.org portal (formally biosharing.org) for metadata standards in the biosciences has a comprehensive curated catalogue [[38](#_ENREF_38)]. The positive response to improving interoperability indicates the community’s need for such standardization[[39](#_ENREF_39)] [)](https://paperpile.com/c/tTHfas/OvwW).

**References**

1. Simonyan V, Goecks J, Mazumder R (2017) Biocompute Objects-A Step towards Evaluation and Validation of Biomedical Scientific Computations. PDA J Pharm Sci Technol 71: 136-146.

2. Sansone SA, Gonzalez-Beltran A, Rocca-Serra P, Alter G, Grethe JS, et al. (2017) DATS, the data tag suite to enable discoverability of datasets. Sci Data 4: 170059.

3. Chapman B (2018) BC-Bio NextGen. In: nextgen b-b, editor. GitHub.

4. Koster J, Rahmann S (2012) Snakemake--a scalable bioinformatics workflow engine. Bioinformatics 28: 2520-2522.

5. Di Tommaso P, Chatzou M, Floden EW, Barja PP, Palumbo E, et al. (2017) Nextflow enables reproducible computational workflows. Nat Biotechnol 35: 316-319.

6. Vivian J, Rao AA, Nothaft FA, Ketchum C, Armstrong J, et al. (2017) Toil enables reproducible, open source, big biomedical data analyses. Nat Biotechnol 35: 314-316.

7. DNAnexus (2018) DNAnexus.

8. Afgan E, Baker D, Coraor N, Goto H, Paul IM, et al. (2011) Harnessing cloud computing with Galaxy Cloud. Nat Biotechnol 29: 972-974.

9. Afgan E, Baker D, van den Beek M, Blankenberg D, Bouvier D, et al. (2016) The Galaxy platform for accessible, reproducible and collaborative biomedical analyses: 2016 update. Nucleic Acids Res 44: W3-W10.

10. Goecks J, Nekrutenko A, Taylor J (2010) Galaxy: a comprehensive approach for supporting accessible, reproducible, and transparent computational research in the life sciences. Genome Biol 11: R86.

11. Simonyan V, Mazumder R (2014) High-Performance Integrated Virtual Environment (HIVE) Tools and Applications for Big Data Analysis. Genes (Basel) 5: 957-981.

12. Simonyan V, Chumakov K, Dingerdissen H, Faison W, Goldweber S, et al. (2016) High-performance integrated virtual environment (HIVE): a robust infrastructure for next-generation sequence data analysis. Database (Oxford) 2016.

13. Peter Amstutz MRC, Nebojša Tijanić (editors), Brad Chapman, John Chilton, Michael Heuer, Andrey Kartashov, Dan Leehr, Hervé Ménager, Maya Nedeljkovich, Matt Scales, Stian Soiland-Reyes, Luka Stojanovic (2016) Common Workflow Language,. Specification, Common Workflow Language working group. .

14. Kaushik G, Ivkovic S, Simonovic J, Tijanic N, Davis-Dusenbery B, et al. (2016) Rabix: An Open-Source Workflow Executor Supporting Recomputability and Interoperability of Workflow Descriptions. Pac Symp Biocomput 22: 154-165.

15. Ison J, Rapacki K, Menager H, Kalas M, Rydza E, et al. (2016) Tools and data services registry: a community effort to document bioinformatics resources. Nucleic Acids Res 44: D38-47.

16. Alterovitz G, Warner J, Zhang P, Chen Y, Ullman-Cullere M, et al. (2015) SMART on FHIR Genomics: facilitating standardized clinico-genomic apps. J Am Med Inform Assoc 22: 1173-1178.

17. Michener WK (2006) Meta-information concepts for ecological data management. Ecological Informatics 1: 3-7.

18. Lawler M, Siu LL, Rehm HL, Chanock SJ, Alterovitz G, et al. (2015) All the World's a Stage: Facilitating Discovery Science and Improved Cancer Care through the Global Alliance for Genomics and Health. Cancer Discovery 5: 1133-1136.

19. Hayden EC (2013) Geneticists push for global data-sharing. Nature 498: 16-17.

20. Siu LL, Lawler M, Haussler D, Knoppers BM, Lewin J, et al. (2016) Facilitating a culture of responsible and effective sharing of cancer genome data. Nature Medicine 22: 464-471.

21. Bechhofer S, Buchan I, De Roure D, Missier P, Ainsworth J, et al. (2013) Why linked data is not enough for scientists. Future Generation Computer Systems-the International Journal of Grid Computing and Escience 29: 599-611.

22. Connett J (2008) Repeatability and Reproducibility, with Applications to Design of Clinical Trials. Wiley Online Library.

23. Hettne KM, Dharuri H, Zhao J, Wolstencroft K, Belhajjame K, et al. (2014) Structuring research methods and data with the research object model: genomics workflows as a case study. J Biomed Semantics 5: 41.

24. Chard K, D'Arcy M, Heavner B, Foster I, Kesselman C, et al. (2016) I'll Take That to Go: Big Data Bags and Minimal Identifiers for Exchange of Large, Complex Datasets. 2016 Ieee International Conference on Big Data (Big Data): 319-328.

25. Gonzalez-Beltran A, Li P, Zhao J, Avila-Garcia MS, Roos M, et al. (2015) From Peer-Reviewed to Peer-Reproduced in Scholarly Publishing: The Complementary Roles of Data Models and Workflows in Bioinformatics. PLoS One 10.

26. Peng RD (2011) Reproducible research in computational science. Science 334: 1226-1227.

27. De Roure D (2011) Towards the preservation of scientific workflows. International Conference on Preservation of Digital Objects UK.

28. Page K PR (2012) From workflows to Research Objects: an architecture for preserving the semantics of science. Procedings of the 2nd International Workshop on Linked Science. Boston, USA.

29. Belhajjame K ZJ, Garijo D, Gamble M, Hettne K, Palma R, Mina, Corcho O, Gomez-Perez J, Bechhofer S, Klyne Graham, Goble C (2015) Using a suite of ontologies for preserving workflow-centric research objects. Web Semantics: Science, Services and Agents on the World Wide Web: pp.16–42.

30. Kaye J, Heeney C, Hawkins N, de Vries J, Boddington P (2009) Data sharing in genomics--re-shaping scientific practice. Nat Rev Genet 10: 331-335.

31. Stein L (2002) Creating a bioinformatics nation. Nature 417: 119-120.

32. Navas-Delgado I, Rojano-Munoz Mdel M, Ramirez S, Perez AJ, Andres Leon E, et al. (2006) Intelligent client for integrating bioinformatics services. Bioinformatics 22: 106-111.

33. Katayama T, Arakawa K, Nakao M, Ono K, Aoki-Kinoshita KF, et al. (2010) The DBCLS BioHackathon: standardization and interoperability for bioinformatics web services and workflows. The DBCLS BioHackathon Consortium*. J Biomed Semantics 1: 8.

34. Wilkinson MD, Links M (2002) BioMOBY: an open source biological web services proposal. Brief Bioinform 3: 331-341.

35. Stajich JE, Lapp H (2006) Open source tools and toolkits for bioinformatics: significance, and where are we? Brief Bioinform 7: 287-296.

36. Ison J, Kalas M, Jonassen I, Bolser D, Uludag M, et al. (2013) EDAM: an ontology of bioinformatics operations, types of data and identifiers, topics and formats. Bioinformatics 29: 1325-1332.

37. Garijo D, Kinnings S, Xie L, Zhang Y, Bourne PE, et al. (2013) Quantifying reproducibility in computational biology: the case of the tuberculosis drugome. PLoS One 8: e80278.

38. McQuilton P, Gonzalez-Beltran A, Rocca-Serra P, Thurston M, Lister A, et al. (2016) BioSharing: curated and crowd-sourced metadata standards, databases and data policies in the life sciences. Database (Oxford) 2016.

39. Gil Y, Deelman E, Ellisman M, Fahringer TF, Fox G, et al. (2007) Examining the challenges of scientific workflows. Computer 40: 24-+.
